# The Construction and Development of a Clinical Prediction Model to Assess Lymph Node Metastases in Osteosarcoma

**DOI:** 10.3389/fpubh.2021.813625

**Published:** 2022-01-06

**Authors:** Wenle Li, Shengtao Dong, Bing Wang, Haosheng Wang, Chan Xu, Kai Zhang, Wanying Li, Zhaohui Hu, Xiaoping Li, Qiang Liu, Rilige Wu, Chengliang Yin

**Affiliations:** ^1^Department of Orthopedics, Xianyang Central Hospital, Xianyang, China; ^2^Clinical Medical Research Center, Xianyang Central Hospital, Xianyang, China; ^3^Department of Spine Surgery, Second Affiliated Hospital of Dalian Medical University, Dalian, China; ^4^Department of Orthopaedics, The Second Hospital of Jilin University, Changchun, China; ^5^Department of Spine Surgery, Liuzhou People's Hospital, Liuzhou, China; ^6^Shulan International Medical College, Zhejiang Shuren University, Hangzhou, China; ^7^College of Information and Electrical Engineering, China Agricultural University, Beijing, China; ^8^Faculty of Medicine, Macau University of Science and Technology, Macau, Macao SAR, China

**Keywords:** nomogram, SEER, osteosarcoma, lymph node metastases, multicenter

## Abstract

**Background:** This study aimed to construct a clinical prediction model for osteosarcoma patients to evaluate the influence factors for the occurrence of lymph node metastasis (LNM).

**Methods:** In our retrospective study, a total of 1,256 patients diagnosed with chondrosarcoma were enrolled from the SEER (Surveillance, Epidemiology, and End Results) database (training cohort, *n* = 1,144) and multicenter dataset (validation cohort, *n* = 112). Both the univariate and multivariable logistic regression analysis were performed to identify the potential risk factors of LNM in osteosarcoma patients. According to the results of multivariable logistic regression analysis, A nomogram were established and the predictive ability was assessed by calibration plots, receiver operating characteristics (ROCs) curve, and decision curve analysis (DCA). Moreover, Kaplan-Meier plot of overall survival (OS) was plot and a web calculator visualized the nomogram.

**Results:** Five independent risk factors [chemotherapy, surgery, lung metastases, lymphatic metastases (M-stage) and tumor size (T-stage)] were identified by multivariable logistic regression analysis. What's more, calibration plots displayed great power both in training and validation group. DCA presented great clinical utility. ROCs curve provided the predictive ability in the training cohort (AUC = 0.805) and the validation cohort (AUC = 0.808). Moreover, patients in LNN group had significantly better survival than that in LNP group both in training and validation group.

**Conclusion:** In this study, we constructed and developed a nomogram with risk factors, which performed well in predicting risk factors of LNM in osteosarcoma patients. It may give a guide for surgeons and oncologists to optimize individual treatment and make a better clinical decision.

## Introduction

Osteosarcoma is a common malignant bone tumor. The primary treatment consisting of neoadjuvant therapy, surgery and postoperative chemotherapy have resulted in the 5-year overall survival rate of ~60% (1, 2). However, even with the treatment of surgery and chemotherapy, the prognosis for patients with metastatic osteosarcoma remains dismal (3, 4).The lung metastases, the primary target of metastasis in osteosarcoma, has five-year survival rates of ~30% (5, 6). In extrapulmonary metastatic osteosarcoma, patients with lymph node metastases (LNM) have worse clinical outcomes, with five-year survival rates of 10% (7). However, only 3% of patients with osteosarcoma are diagnosed with LNM, leading to the lack of adequate clinical data for exploring osteosarcoma LNM (8).Therefore, a population-based study to assess the LNM in osteosarcoma is imminent.

Furthermore, disease forecasting is a vital part of the medical research (9–18). As a visual prediction tool, nomogram lists each variable separately and assigns a corresponding score for each status (19). Based on these considerations, we mined the Surveillance, Epidemiology, and End Results (SEER) database to construct the nomogram and used data from four academic hospitals for independent validation. This study contributes to providing more personalized guidance for patient care and improving patients' prognosis.

## Materials and Methods

### Data Collection

In the present study, patients diagnosed with osteosarcoma between 2010 and 2016 were collected. The training group were extracted from the Surveillance, Epidemiology, and End Results (SEER) database with the SEER ^*^ Stat software version 8.3.6. And the third edition of the International Taxonomy of Oncology (ICDO-3), morphological code (9220) was used to identify osteosarcoma. The exclusion criteria of the training group were as follows: (1) patients with no positive pathology; (2) patients with unknown lymph node status and survival time; (3) more than one primary tumor.

Data of the validation group were obtained from four academic institutions, the Second Affiliated Hospital of Jilin University, the Second Affiliated Hospital of Dalian Medical University, the Liuzhou People's Hospital affiliated to Guangxi Medical University, and the Xianyang Central Hospital. And during the period of investigation, each center was responsible for the acquisition of data by three investigators. Two investigators were responsible for data extraction and the accuracy check was conducted by the third investigator. The exclusion criteria were consistent with the training group. For multicenter data, the study was approved by the ethics review committee of four medical institutions in China, the Second Affiliated Hospital of Jilin University, the Second Affiliated Hospital of Dalian Medical University, Liuzhou People's Hospital, and Xianyang Central Hospital (No. 2021-00-22) and was conducted in accordance with the guidelines of the Helsinki Declaration.

Demographic and clinical variables, including race, age, survival time, sex, primary site, grade, laterality, tumor size (T-stage), distance metastases (M-stage), surgery, radiation, chemotherapy, bone metastases and lung metastases were considered in this study.

### Statistical Analysis

Continuous variables were expressed as mean ± standard deviation (SD), and categorical variables were expressed as frequency (proportions). The Student's *t*-test, Chi-square tests and Mann–Whitney tests were applied to continuous variables and categorical variables respectively with IBM SPSS Statistics version 26.0 (SPSS Inc., Chicago, Illinois, USA). Risk factors of the osteosarcoma were assessed using logistic regression. All analyzes were performed using R software version 3.6.2 (http://www.r-project.org/) including multiple R packages (Including regplot, rms, rmda and pROC). *P* values < 0.05 were considered statistically significant, and confidence intervals (CIs) were expressed as 95% confidence levels.

### Construction, Validation and Clinical Utility of a Nomogram

The following variables were included in the univariate logistic regression analysis: race, age, survival time, sex, primary site, grade, laterality, tumor size, lymphatic metastasis, surgery, radiation, chemotherapy, bone metastases and lung metastases. According to the result of the univariate logistic regression analysis with the *P* value < 0.05, we performed the multivariable logistic regression analysis. And the Nomogram was constructed based on the results of multivariable logistic regression analysis with the *P* value < 0.05. Calibration plot and receiver operating characteristic (ROC) curves were used to evaluate the prediction performance of the nomogram. The higher the area under the curves (AUC) of ROC indicated the better model performance. In addition, the decision curve analysis (DCA) was used to evaluate the clinical utility of nomogram in decision-making. Based on the established nomogram, an interactive convenient web calculator was provided (https://drliwenle.shinyapps.io/LMOOapp/).

## Results

### Demographic Baseline Characteristics

As shown in Table 1, a total of 1,256 patients were enrolled. There was no statistically significant difference between the training group (*n* = 1,144) and validation group (*n* = 112) except the Race (*P* < 0.001) and Chemotherapy (*P* = 0.017).

**Table 1:** Baseline data table of the training group and the validation group.

**Variable**	**level**	**Overall** **(*N* = 1,256)**	**SEER data (Training group, *N* = 1,144)**	**Multicenter data (validation group, *N* = 112)**	***p***
Race (%)	Black	168 (13.4)	168 (14.7)	0 (0.0)	<0.001
	Other	228 (18.2)	116 (10.1)	112 (100.0)	
	White	860 (68.5)	860 (75.2)	0 (0.0)	
Age [mean (SD)]	NA	33.31 (24.31)	33.47 (24.26)	31.62 (24.88)	0.443
Times [mean (SD)]	NA	29.93 (22.69)	29.91 (22.54)	30.10 (24.24)	0.933
Sex (%)	Female	573 (45.6)	521 (45.5)	52 (46.4)	0.936
	Male	683 (54.4)	623 (54.5)	60 (53.6)	
Primary.Site (%)	Axis bone	336 (26.8)	309 (27.0)	27 (24.1)	0.349
	Limb bone	817 (65.0)	738 (64.5)	79 (70.5)	
	Other	103 (8.2)	97 (8.5)	6 (5.4)	
Grade (%)	Moderately differentiated	41 (3.3)	41 (3.6)	0 (0.0)	0.124
	Poorly differentiated	302 (24.0)	279 (24.4)	23 (20.5)	
	Undifferentiated; anaplastic	560 (44.6)	511 (44.7)	49 (43.8)	
	Unknown	324 (25.8)	287 (25.1)	37 (33.0)	
	Well-differentiated	29 (2.3)	26 (2.3)	3 (2.7)	
Laterality (%)	Left	537 (42.8)	494 (43.2)	43 (38.4)	0.08
	Not a paired site	173 (13.8)	163 (14.2)	10 (8.9)	
	Right	546 (43.5)	487 (42.6)	59 (52.7)	
T (%)	T1	426 (33.9)	388 (33.9)	38 (33.9)	0.294
	T2	569 (45.3)	523 (45.7)	46 (41.1)	
	T3	42 (3.3)	35 (3.1)	7 (6.2)	
	TX	219 (17.4)	198 (17.3)	21 (18.8)	
M (%)	M0	976 (77.7)	892 (78.0)	84 (75.0)	0.547
	M1	280 (22.3)	252 (22.0)	28 (25.0)	
Surgery (%)	No	254 (20.2)	230 (20.1)	24 (21.4)	0.834
	Yes	1002 (79.8)	914 (79.9)	88 (78.6)	
Radiation (%)	No	1103 (87.8)	999 (87.3)	104 (92.9)	0.119
	Yes	153 (12.2)	145 (12.7)	8 (7.1)	
Chemotherapy (%)	No	274 (21.8)	260 (22.7)	14 (12.5)	0.017
	Yes	982 (78.2)	884 (77.3)	98 (87.5)	
Bone metastases (%)	No	1146 (91.2)	1044 (91.3)	102 (91.1)	0.981
	Unknown	52 (4.1)	47 (4.1)	5 (4.5)	
	Yes	58 (4.6)	53 (4.6)	5 (4.5)	
Lung metastases (%)	No	988 (78.7)	901 (78.8)	87 (77.7)	0.931
	Unknown	48 (3.8)	44 (3.8)	4 (3.6)	
	Yes	220 (17.5)	199 (17.4)	21 (18.8)	

Additionally, these patients were divided into two subgroups according to the LNM in Table 2. There were no significant differences in race, sex, laterality and radiation between the lymph node negative group (LNN, *n* = 1,104) and the lymph node positive (or unable to evaluate) group (LNP, *n* = 152). However, the other characteristics showed significant differences between the two groups.

**Table 2:** Patient baseline table of lymphatic metastases.

	**Level**	**Overall (*N* = 1,256)**	**No (*N* = 1,104)**	**Yes/Unable to evaluate (*N* = 152)**	***p***
Category (%)	Multicenter data (validation group)	112 (8.9)	93 (8.4)	19 (12.5)	0.133
	SEER data (Training group)	1144 (91.1)	1011 (91.6)	133 (87.5)	
Times [mean (SD)]	NA	29.93 (22.69)	30.95 (22.70)	22.45 (21.18)	<0.001
Race (%)	black	168 (13.4)	145 (13.1)	23 (15.1)	0.417
	Other	228 (18.2)	196 (17.8)	32 (21.1)	
	White	860 (68.5)	763 (69.1)	97 (63.8)	
Age [mean (SD)]	NA	33.31 (24.31)	32.48 (23.79)	39.35 (27.16)	0.001
Sex (%)	Female	573 (45.6)	498 (45.1)	75 (49.3)	0.37
	Male	683 (54.4)	606 (54.9)	77 (50.7)	
Primary.Site (%)	Axis bone	336 (26.8)	282 (25.5)	54 (35.5)	0.027
	Limb bone	817 (65.0)	732 (66.3)	85 (55.9)	
	Other	103 (8.2)	90 (8.2)	13 (8.6)	
Grade (%)	Moderately differentiated	41 (3.3)	39 (3.5)	2 (1.3)	<0.001
	Poorly differentiated	302 (24.0)	273 (24.7)	29 (19.1)	
	Undifferentiated; anaplastic	560 (44.6)	508 (46.0)	52 (34.2)	
	Unknown	324 (25.8)	257 (23.3)	67 (44.1)	
	Well-differentiated	29 (2.3)	27 (2.4)	2 (1.3)	
Laterality (%)	Left	537 (42.8)	483 (43.8)	54 (35.5)	0.104
	Not a paired site	173 (13.8)	146 (13.2)	27 (17.8)	
	Right	546 (43.5)	475 (43.0)	71 (46.7)	
T (%)	T1	426 (33.9)	400 (36.2)	26 (17.1)	<0.001
	T2	569 (45.3)	526 (47.6)	43 (28.3)	
	T3	42 (3.3)	36 (3.3)	6 (3.9)	
	TX	219 (17.4)	142 (12.9)	77 (50.7)	
M (%)	M0	976 (77.7)	883 (80.0)	93 (61.2)	<0.001
	M1	280 (22.3)	221 (20.0)	59 (38.8)	
Surgery (%)	No	254 (20.2)	179 (16.2)	75 (49.3)	<0.001
	Yes	1002 (79.8)	925 (83.8)	77 (50.7)	
Radiation (%)	No	1103 (87.8)	975 (88.3)	128 (84.2)	0.187
	Yes	153 (12.2)	129 (11.7)	24 (15.8)	
Chemotherapy (%)	No	274 (21.8)	208 (18.8)	66 (43.4)	<0.001
	Yes	982 (78.2)	896 (81.2)	86 (56.6)	
Bone metastases (%)	No	1146 (91.2)	1046 (94.7)	100 (65.8)	<0.001
	Unknown	52 (4.1)	14 (1.3)	38 (25.0)	
	Yes	58 (4.6)	44 (4.0)	14 (9.2)	

### Univariate and Multivariable Logistic Regression Results

According to the univariate logistics regression analysis, we identified 10 prognostic factors including age, survival time, primary site, laterality, tumor size, lymph metastasis, surgery, chemotherapy, bone metastases and lung metastases in the training set (*P* < 0.05) (Table 3). Then, based on the above result, applying the multivariable logistics regression analysis, we figured out five independent prognostic factors including T-stage [TX: odds ratio (OR) 3.602,95%CI 1.710–5.483, *P* < 0.001], M-stage (M1: OR = 2.890, 1.463–5.709, *P* < 0.01), surgery (Yes: OR = 0.418, 0.247–0.706, *P* < 0.01), Chemotherapy (Yes: OR = 0.475, 0.267–0.819, *P* < 0.01)and Lung metastases (Unknown: OR = 9.407, 1.955–45.261, *P* < 0.01) (Table 3).

**Table 3:** Univariate and multifactorial logistic regression analysis of risk factors for Lymph node metastases in patients with osteosarcoma.

**Variables**	**Univariate OR (95% CI)**	**p value**	**Multivariate OR (95% CI)**	***p*** **value**
Age (years)	1.011 (1.004–1.018)	<0.01	0.993 (0.982–1.004)	0.397
Survival time (month)	0.983 (0.974–0.992)	<0.001	0.998 (0.987–1.010)	0.788
**Race**				
White	Reference	Ref	Ref	Ref
Black	1.248 (0.766–3.033)	0.374	/	/
Other	0.993 (0.537–1.835)	0.982	/	/
**Sex**				
Male	Ref	Ref	Ref	Ref
Female	1.163 (0.810–1.671)	0.412	/	/
**Primary site**				
Limb bones	Ref	Ref	Ref	Ref
Axis of a bone	1.675 (1.133–2.478)	<0.05	0.946 (0.517–1.731)	0.857
Other	1.286 (0.671–2.466)	0.449	1.840 (0.806–4.198)	0.148
**Grade**				
Well-differentiated	Ref	Ref	Ref	Ref
Moderately differentiated	1.282 (0.110–14.893)	0.843	/	/
Poorly differentiated	2.569 (0.334–19.741)	0.364	/	/
Undifferentiated; anaplastic	2.473 (0.328–18.673)	0.380	/	/
Unknown	6.332 (0.840–47.705)	0.073	/	/
**Laterality**				
Left	Ref	Ref	Ref	Ref
Right	1.395 (0.930–2.091)	0.108	1.477 (0.920–2.371)	0.106
Other	1.848 (1.102–3.101)	<0.05	1.113 (0.536–2.310)	0.774
**T**				
T1	Ref	Ref	Ref	Ref
T2	1.121 (0.657–1.912)	0.675	0.960 (0.540–1.707)	0.889
T3	2.528 (0.900–7.101)	0.078	1.473 (0.476–4.559)	0.502
TX	7.933 (4.780–13.167)	<0.001	3.062 (1.710–5.483)	<0.001
**M**				
M0	Ref	Ref	Ref	Ref
M1	2.506 (1.710–3.673)	<0.001	2.890 (1.463–5.709)	<0.01
**Surgery**				
No	Ref	Ref	Ref	Ref
Yes	0.197 (0.135–0.287)	<0.001	0.418 (0.247–0.706)	<0.01
**Radiation**				
No	Ref	Ref	Ref	Ref
Yes	1.524 (0.936–2.481)	0.091	/	/
**Chemotherapy**				
No	Ref	Ref	Ref	Ref
Yes	0.289 (0.199–0.421)	<0.001	0.475 (0.276–0.819)	<0.01
**Bone metastases**				
No	Ref	Ref	Ref	Ref
Yes	3.260 (1.652–6.436)	<0.01	1.257 (0.570–2.772)	0.571
Unknown	32.490 (16.267–64.892)	<0.001	2.159 (0.483–9.659)	0.314
**Lung metastases**				
No	Ref	Ref	Ref	Ref
Yes	2.938 (1.887–4.573)	<0.001	0.902 (0.449–1.812)	0.771
Unknown	45.225 (21.360–95.754)	<0.001	9.407 (1.955–45.261)	<0.01

### Construction and Validation of Nomogram for Chondrosarcoma Patients

**Figure 1:**
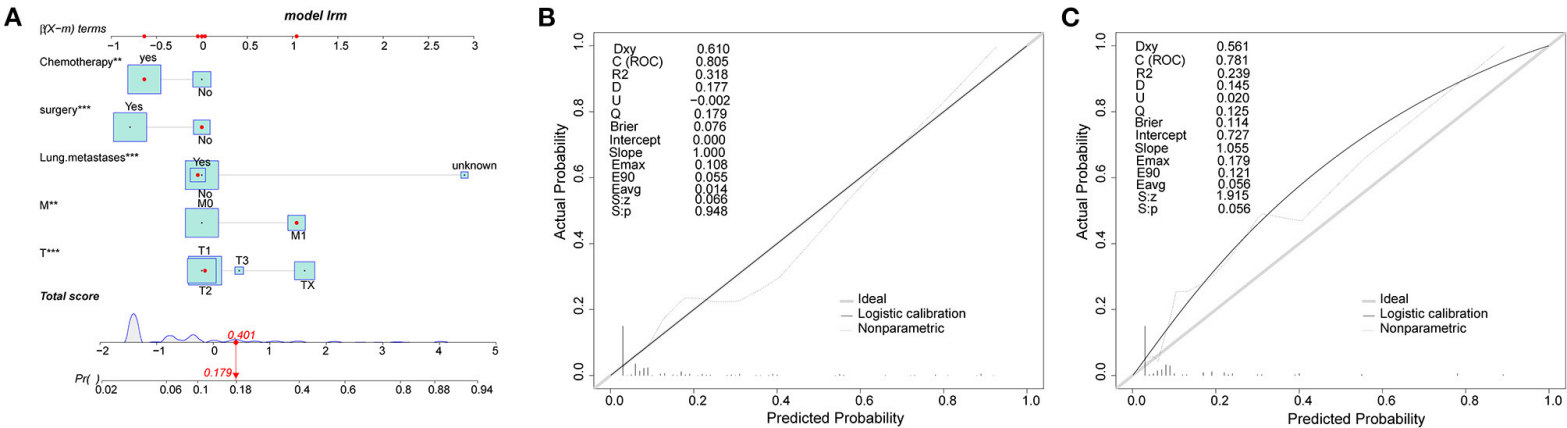
**(A)** Nomogram for osteosarcoma patients. **(B,C)** are training cohorts and the validation cohorts calibration diagram respectively, which indicate good consistency.

**Figure 2:**
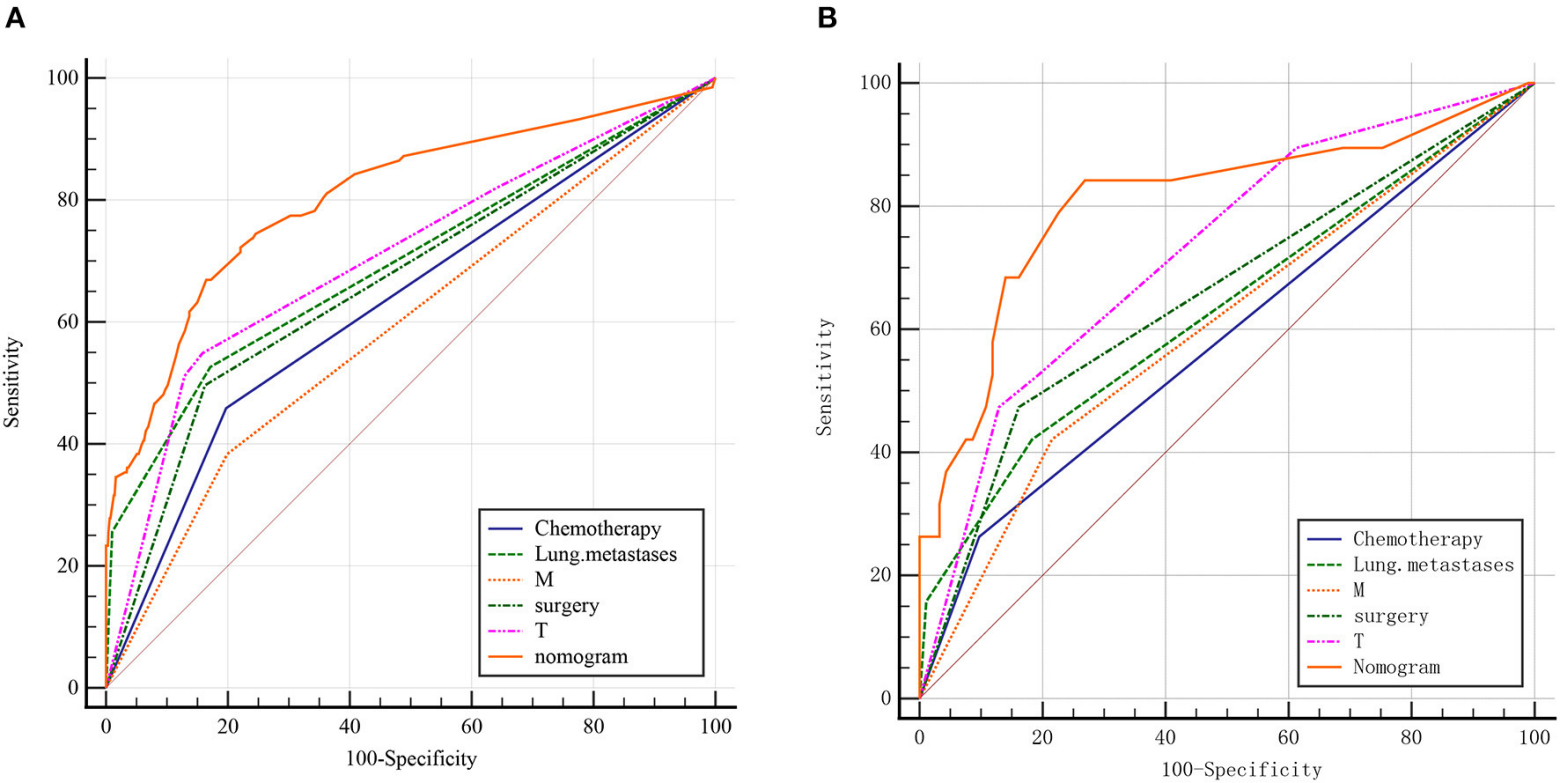
ROC curves for the training and validation group. **(A)** training group; **(B)** validation group.

The nomogram contained five risk factors confirmed to be statistically significant by logistic regression analysis, including T-stage, M-stage, surgery, chemotherapy and lung metastases (Figure 1A). Calibration chart of nomogram showed a good consistency in the training and validation groups (Figures 1B,C). The AUC values of nomogram were 0.805 (95% CI 0.781–0.827) and 0.808 (95% CI 0.723–0.877) in the training group and the validation group respectively (Figures 2A,B). Furthermore, the ROC curve demonstrated a superior performance of the nomogram compared to the single variable, including chemotherapy (AUC = 0.631, 95%CI 0.602 to 0.659), lung metastases (0.697, 0.669 to 0.723), M-stage (AUC = 0.592, 95%CI 0.563 to 0.621), surgery (AUC = 0.667, 95%CI 0.639 to 0.694) and T-stage (0.706, 95%CI 0.678 to 0.732). The statistical results of validation group were consistent with the training group as shown in Table 4. In addition, an online web calculator was designed (https://drliwenle.shinyapps.io/LMOOapp/).

**Table 4:** AUC of training group and validation group.

	**SEER data** **(Training group)**			**Multicenter data** **(validation group)**		
**Variable**	**AUC**	**SE**	**95% CI**	**AUC**	**SE**	**95% CI**
Chemotherapy	0.631	0.0226	0.602 to 0.659	0.583	0.0541	0.486 to 0.676
Lung metastases	0.697	0.0239	0.669 to 0.723	0.631	0.0644	0.535 to 0.721
M	0.592	0.0221	0.563 to 0.621	0.603	0.062	0.506 to 0.694
Surgery	0.667	0.0225	0.639 to 0.694	0.656	0.0619	0.561 to 0.743
T	0.706	0.0256	0.678 to 0.732	0.728	0.0614	0.635 to 0.807
Nomogram	0.805	0.0231	0.781 to 0.827	0.808	0.065	0.723 to 0.877

### Clinical Applicability of the Nomogram

**Figure 3:**
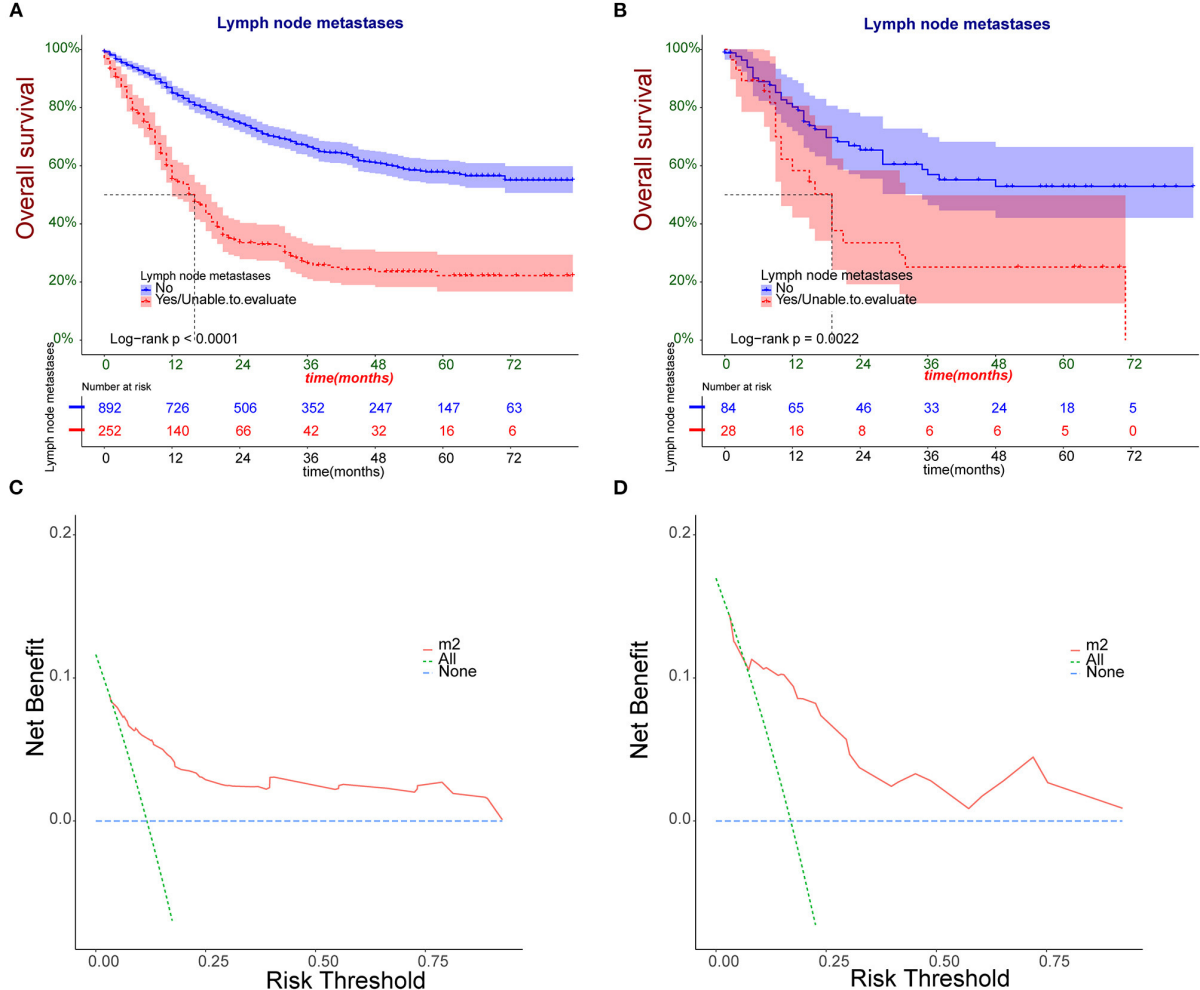
**(A,B)** The Kaplan-Meier survival analysis patients with osteosarcoma in training and validation group. **(C,D)** Nomogram decision curve (DCA) of the training and validation group.

The Kaplan-Meier survival curves of training group were plotted (Figure 3A). The results revealed that the overall survival (OS) significantly decreased in patients with LNP comparing with the LNN (*P* < 0.001). Moreover, the threshold about 0.1 to 0.9 had the maximum benefit range of the model as shown in the DCA curve (Figures 3C,D). The Kaplan-Meier survival curves of validation group displayed the same trend between the two groups (P <0.001) (Figure 3B).

## Discussion

Osteosarcoma metastases, which are typically secondary to hematogenous dissemination and the occurrence of lymph system is extremely rare, have been identified to be significantly associated with poor prognosis (1–4, 7, 20, 21). Comparing to the most common lung metastases, osteosarcoma patients with lymph node involvement have a worse prognosis, suggesting that the invasion of the lymph nodes is an important indicator for the assessment of malignancy stage and the selection of a correct treatment protocol (22, 23).Meanwhile, studies points out that FDG-PET and ^99m^Tc-labeled biomineralization nanoprobe are effective in early diagnosis of metastatic lymph nodes in osteosarcoma (24–27). Therefore, real-time lymph node surveillance and radical treatment for osteosarcoma patients with a high risk of lymph node metastasis will improve patient survival (8). In this study, we identified five independent risk factors associated with LNM and provided a convenient nomogram prediction model and a web calculator on the basis of the model.

Surgery and chemotherapy are the most reliable and effective treatment options for prolonging the lives of patients and have been followed in several clinical trials (28–30). According to the multivariable logistic analysis, chemotherapy and surgery were crucial prognostic indicators. Surgery and chemotherapy contributed to improve patient prognosis, which were consistent with the result of the KM survival curve. This suggests that timely and effective treatment plays an important role in controlling lymph node metastasis and enhancing OS (15, 17, 31, 32). Unfortunately, the relationship between radiotherapy and lymph node metastasis has not been proved. A possible reason for these results would be the uncertainty of radiation therapy in cancer treatment along with the development of radiation-associated osteosarcoma make the safety of chemotherapy need to be further ensured (33, 34).

Due to the lack of lymphatic drainage in normal cortical bone and spongy bone, LNM is rare in bone sarcomas (35, 36).Regional lymph node involvement in osteosarcoma may be owing to the infiltration of the enlarged tumor parenchyma into the periphery, such as the joint capsule or synovium, leading to dissemination into the lymphatic system (37, 38).Our study reported T-stage as a significant predictor of LNM, which was also consistent with previous findings (7).Meanwhile, because of the fact that the peak incidence of the osteosarcoma is 15–19 years of age, more than 80% of patients achieve limb salvage through surgery (4, 28, 38).In this respect, the surgeons need pay more attention to the status of the regional lymph nodes during the resection of larger osteosarcoma in order to eradicate the sarcoma and preserve the limb. M-stage indicates distant metastasis in osteosarcoma, and it is also a risk factor associated with LNM. In the study by Thampi et al. osteosarcoma lymphatic metastasis was significantly associated with distant metastasis (7).Furthermore, since the lung was the most important target organ for distant metastasis in osteosarcoma, we separated this variable from distant metastasis to emphasize the significant role played by lung metastasis in assessing lymph node status. Patients with lung metastases express the characteristic biomarker, such as KEAP, Matrix-Gla and Rab22a (39–41). Considering the intense correlation we found between lung metastasis and lymph node involvement, these reported biomarkers would be triggers for LNM. Therefore, this provides inspiration to further molecular biology studies focusing on lymph node metastasis in osteosarcoma.

We constructed a novel nomogram to assess the risk of developing lymph node metastasis in osteosarcoma, and the discriminatory of any individual predictor was inferior to that of nomogram, suggesting that the nomogram model indicated promising prospects for tumor surveillance and clinical decision making. Although some predictive nomograms have been reported in previous studies, our study complements previous work. Compared to Dong et al.'s study, external independent validation consisting of multiple academic centers is a prominent feature of this study, and the inclusion of multiple ethnic groups enhances the credibility of the results (8). Moreover, we provide a convenient and digital prediction tool for users. By analyzing the clinical characteristics and associated risk factors, we improve the prediction of lymph node metastasis risk in osteosarcoma and provide a basis for individualized treatment and follow-up strategies. The web-based calculator constructed in this study is an easy-to-use clinical tool that helps to promote personalized treatment.

Finally, one obvious limitation in this study was that the statistically significant difference between the training group and validation groups in chemotherapy may have an influence on the results. Another limitation was the lack of a more thoroughly analysis due to the inadequacy of systematic and prospective data.

## Conclusion

In conclusion, we constructed a novel nomogram model to predict risk factors for osteosarcoma patients developing LNM, including T-stage, M-stage, surgery, chemotherapy and lung metastases based on epidemiological characteristics obtained from the SEER database and the multicenter hospitals. By combining DCA curve, ROC curve, KM curve, web calculator and external validation, our nomogram provided an accurate assessment for individualized risk of lymph node metastasis which was helpful for clinicians to make better surgery decisions.

## Data Availability Statement

The raw data supporting the conclusions of this article will be made available by the authors without undue reservation.

## Ethics Statement

The SEER database is a comprehensive data source developed based on population data and updated annually since its launch in 1973. It is public and identifiably accessible that data analysis is treated as non-human subjects by the Office for Human Research Protections. As such, no institutional review board approval and informed consent were required. For multicenter data, the study was approved by the ethics review committee of four medical institutions in China, the Second Affiliated Hospital of Jilin University, the Second Affiliated Hospital of Dalian Medical University, 291 Liuzhou People's Hospital, and Xianyang Central Hospital (No. 2021-00-22) and was conducted in accordance with the guidelines of the Helsinki.

## Author Contributions

CY, RW, QL, and XL designed the study. WeL and SD performed the study, analyzed the data, and wrote the manuscript. BW and HW provided the expert consultations and clinical suggestions. CX and KZ conceived of the study, participated in its design and coordination. WaL and ZH helped to draft the manuscript. All authors reviewed the final version of the manuscript.

## Conflict of Interest

The authors declare that the research was conducted in the absence of any commercial or financial relationships that could be construed as a potential conflict of interest.

## Publisher's Note

All claims expressed in this article are solely those of the authors and do not necessarily represent those of their affiliated organizations, or those of the publisher, the editors and the reviewers. Any product that may be evaluated in this article, or claim that may be made by its manufacturer, is not guaranteed or endorsed by the publisher.

## Abbreviations

SEER: Surveillance, Epidemiology, and End Results database
ROC: receiver operating characteristic
DCA: decision curve analysis
CI: confidence interval
AUC: area under the curve
LNN: lymph node negative
LNM: lymph node metastases
ICD: International Classification of Diseases
SD: standard deviation
KM curves: Kaplan-Meier curves
OR: odds ratio.
